# Supplementary figures and images for: Cellular production of a counterfeit viral protein confers immunity to infection by a related virus
Source: PeerJ. 2018 Sep 28;6:e5679. doi: 10.7717/peerj.5679 (PMC6166632; doi:10.7717/peerj.5679)

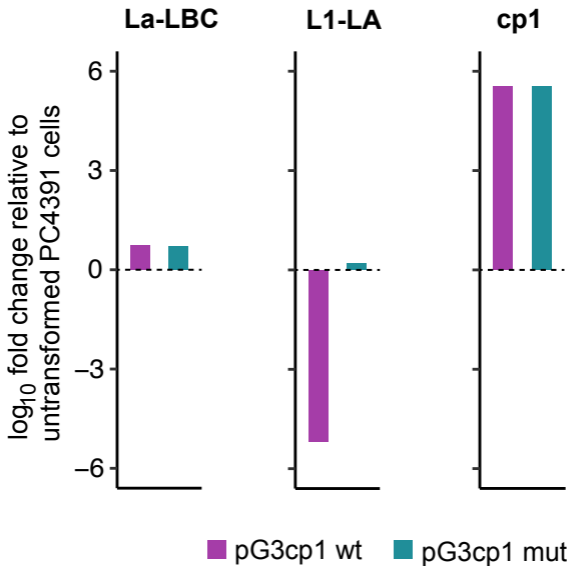

Supplement: File S1 — Relative detection of La–LBC, L1–LA, and cp1 transcripts and genomic RNA by quantitative reverse transcription PCR. PC4391 cells were transformed with pG3cp1 encoding a wild type or a mutated cp1 gene. The pG3cp1 mutant sequence differs from the wild type optimized sequence by a single base (AAG to TAG in codon 13). Abundances are plotted as log10-transformed fold changes relative to untransformed PC4391 cells. L1-LA targets were not detected in pG3cp1 wt treated cells; therefore the maximum Ct value of 40 was conservatively used for analysis. The horizontal dashed lines at zero serve as a reference for detection levels equivalent to those of the untransformed control. Actin was used as a reference gene. All RNA samples were treated with DNase prior to use. [file peerj-06-5679-s001.pdf]

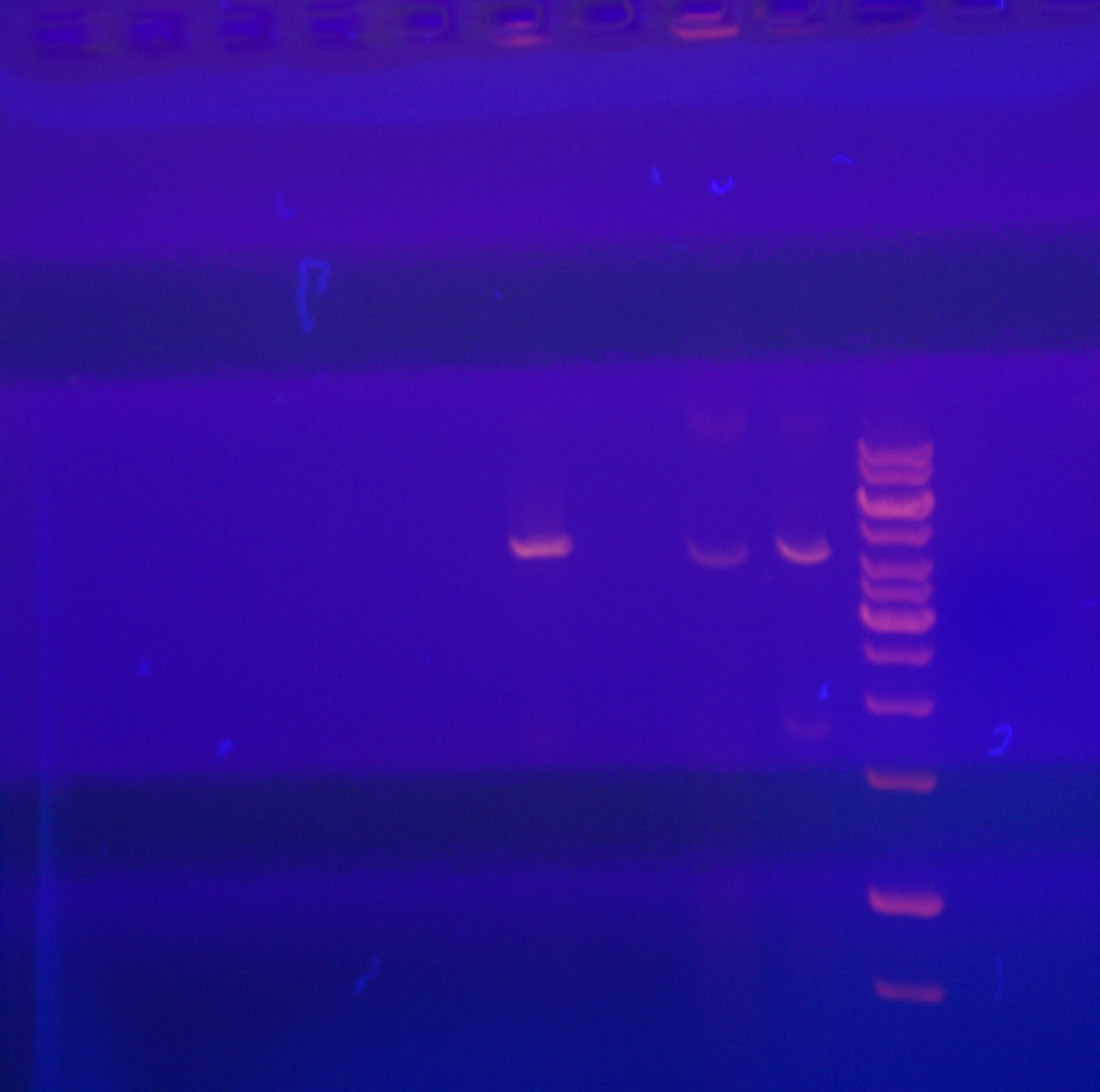

Supplement: Supplemental Information 1 — Total RNA from pYescp1, pYescp1mutant, pG3cp1, pG3cp1mutant, PC847, and markers. [file peerj-06-5679-s003.png]

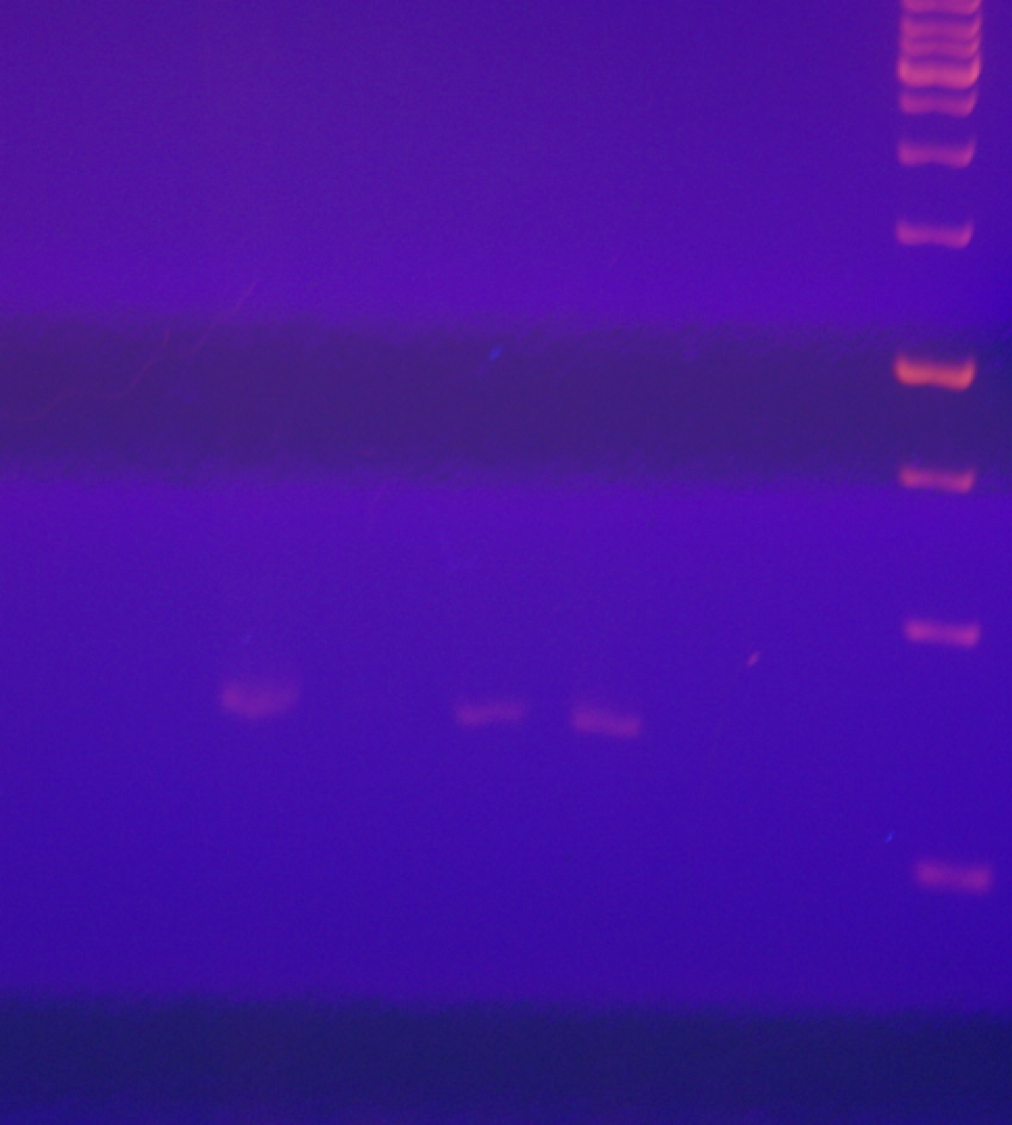

Supplement: Supplemental Information 2 — RTPCR with L1 RdRP primers of pYescp1, pYescp1 mutant, pG3cp1, pG3cp1mutant, and PC847 total RNAs [file peerj-06-5679-s004.png]

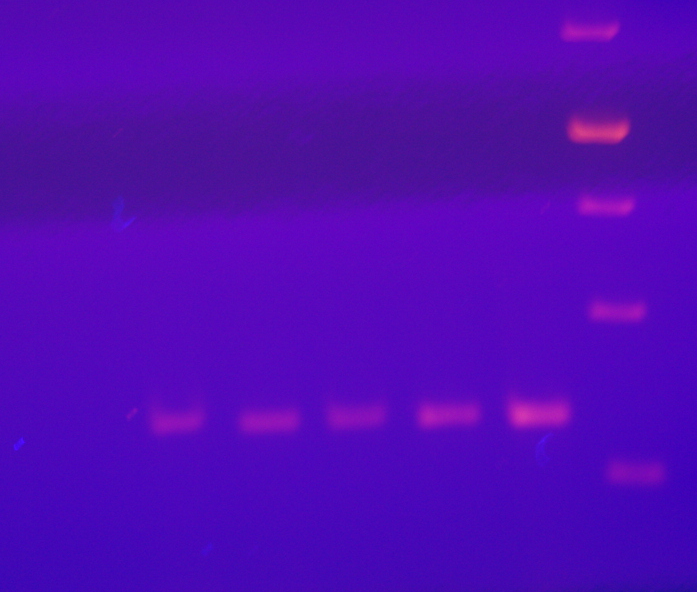

Supplement: Supplemental Information 3 — RTPCR with rps11b primers of pYescp1, pYescp1mutant, pG3cp1, pGccp1mutant, and PC847 total RNA. [file peerj-06-5679-s005.png]

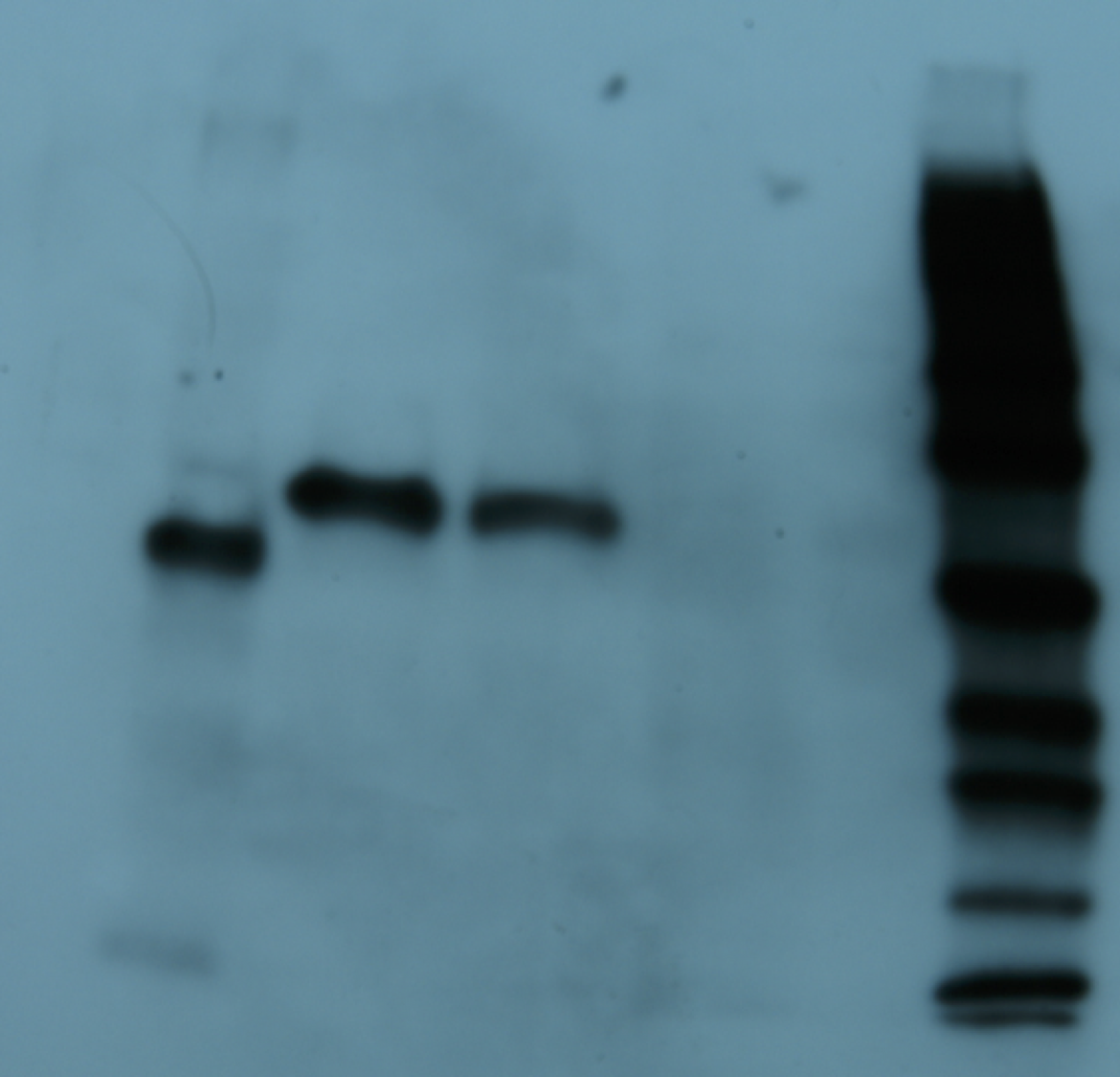

Supplement: Supplemental Information 4 — Figure 3 showing markers. [file peerj-06-5679-s006.png]
