# Supplementary material for: Cellular production of a counterfeit viral protein confers immunity to infection by a related virus
Source: PeerJ. 2018 Sep 28;6:e5679. doi: 10.7717/peerj.5679 (PMC6166632; doi:10.7717/peerj.5679)
Supplement: File S2 — Tryptic peptides are highlighted in bold. [file peerj-06-5679-s002.pdf]

Tryptic peptides detected by GeLC-MS/MS (bold face in sequence)

ScVL1cap

1 MLRFVTKNSQ DKSSDLFSIC SDR**GTFVAHN RVRTDFKFDN LVFNR**VYGVS

51 QKFTLVGNPT VCFNEGSSYL EGIAK**KYLTL DGGLAIDNVL NELR**STCGIP

101 GNAVASHAYN ITSWR**WYDNH VALLMNMLRA YHLQVLTEQG QYSAGDIPMY**

151 **HDGHVKIKLP VTIDDTAGPT QFAWPSDR**ST DSYPDWAQFS ESFPSIDVPY

201 LDVRPLTVTE VNFVLMMMSK WHR**RTNLAID YEAPQLADKF AYRHALTVQD**

251 **ADEWIEGDRT DDQFRPPSSK VMLSALR**KYV NRNRLYNQFY TAAQLLAQIM

301 MKPVPNCAEG YAWLMHDALV NIPKFGSIRG RYPFLLSGDA ALIQATALED

351 WSAIMAKPEL VFTYAMQVSV ALNTGLYLRR VKKTGFGTTI DDSYEDGAFL

401 QPETFVQAAL ACCTGQDAPL NGMSDVYVTY PDLLEFDAVT QVPITVIEPA

451 GYNIVDDHLV VVGVPVACSP YMIFPVAAFD TANPYCGNFV IKAANKYLR**K**

501 **GAVYDKLEAW K**LAWALR**VAG YDTHFKVHGD THGLTK**FYAD NSDTWTHIPE

551 FVTDGDVMEV FVTAIERRAR **HFVELPRLNS PAFFRSVEVS TTIYDTHVQA**

601 **GAHSVYHARR INLDYVKPVS TGIQVINAGE LK**NYWGSVR**R TQQGLGVVGL**

651 TMPAVMPTGE PTAGAAHEEL IEQADNVLVE

Previous mass spec of ScVL1 was also missing internal tryptic peptides between 250 and 500. The situation is similar for DhVcp1 (see below).

DhVcp1his tagged

1 MLKFVNELNS KRKNCLYHSQ QVDGTVRAMS RIRVDFKYNG LKFSR**TLTAS**

51 **QDYTWVGK**AL VCMSEAASSL DGLNKK**YLTL DGAISTENVF QELK**NLAGLQ

101 SNIIAEHTHN VAGWR**WYDNH VALLVNLLRF YILSDLDER**S KLSTGKFPVY

151 DDGHVIIDLN DTLLLEDKAV DWTWPGRRVD ESYPYWNPMT EFLPVTDDPH

201 IDLRPLTEEE AKVVLMMTGE WKPQTNYKLD FYTPRLAEKI MYR**YRNPISS**

251 **LNEWLDAEGT APTYYLPK**SR VIWSALRKYV THNNLYNQFY TATNIVAQVM

301 LTVYPDTAEG MTWLTHVPEV HLPKFGSVRG RYPFLNSGEA AFIQAKALED

351 WAALIAKPEL LFTYGMMLAS TLNIGLAVRD AKASLLIGED KSSFDDTLFL

401 TPETFFASAV SLATGLDAPL NGMGDVYVFY PELVNINETW EVPAVILEPN

451 GYLIKDNHIL STGIPFVGSP YLVYSLAVFD EANPYSGNFV LPEPLRRTRK

501 GAIYSFVDAW KMGWAARIAG YDLSINVFSS NVNYTKYFSP NNNSWSHVLT

551 NGIDDKVEGV LIKDMTRR**SR HFVDLPNFFV PGNHPVTEVK VNVLGTSVLD**

601 **AAGNK**NRAAG TANEWVTPSS LGLQIVSKED VRRFWGHIKR HKSGLAMEGL

651 TMSVNVPAIE GNRGVEVMKG ELR**GHPFEGK PIPNPLLGLD STRTGHHHHH**

701 **H**
